# Supplementary material for: Genetic regions affecting the replication and pathogenicity of dengue virus type 2
Source: PLoS Negl Trop Dis. 2024 Jan 8;18(1):e0011885. doi: 10.1371/journal.pntd.0011885 (PMC10798627; doi:10.1371/journal.pntd.0011885)
Supplement: S1 Table — (PDF) [file pntd.0011885.s001.pdf]

S1 Table. Primer list

Name	Sequence	Melting temperature(℃)
Ribozyme-F	AGGGTCGGCATGGCATCTCCA	75.1
CMVpro-R	CCGGTTCACTAAACCAGCTCT	64.3
DV2-3UTR-R	AGAACCTGTTGATTCAACAGCACCA	69.5
pCMV25-DV2-5UTR-F	AAGCAGAGCTGGTTTAGTGAACCGGAGTTGTTAGTCTACGTGGAC	80.8
DV2-005-Elast-F	GTATTTGGGAGTCATGGTGCAGGCC	73.5
DV2-005-Elast-R	GGCCTGCACCATGACTCCCAAATAC	73.5
DV2-005-NS2Blast-F	CCTGTGGGAAGTGAAGAAACAACGG	72.0
DV2-005-NS2Blast-R	CCGTTGTTTCTTCACTTCCCACAGG	72.0
DV2-005-NS3last-F	CAAAGAATTTGCAGCCGGAAGAAAG	70.8
DV2-005-NS3last-R	CTTTCTTCCGGCTGCAAATTCTTTG	70.8
DV2-005-NS4Blast-F	GAAGAACACAACCAACACAAGAAGG	66.6
DV2-005-NS4Blast-R	CCTTCTTGTGTTGGTTGTGTTCTTC	66.6
pCMV-16681-3UTR25-R	GAGGTGGAGATGCCATGCCGACCCTAGAACCTGTTGATTCAACAG	86.3
DV2-026-NS3last-F	CAAGGAATTCGCAGCTGGAAGAAAA	71.4
DV2-026-NS4Blast-F	GAAGAACACGGCCAACACAAGAAGG	72.1
DV2-026-NS4Blast-R	CCTTCTTGTGTTGGCCGTGTTCTTC	72.1
EcoRV-BIDI-005-SRIP-F	TGGAATTCTGCAGATATCACCATGAATAGGAGACGCAGGTCTGC	83.5
EcoRV-BIDI-026-SRIP-F	TGGAATTCTGCAGATATCACCATGAACAGGAGACGCAGAACTGC	84.4
KpnI-BIDI-005-SRIP-R	AAACGGGCCCGGTACCTCAGGCCTGCACCATGACTCCCA	90.3
Seq1	GGCGTTCCATYTRACCACAC	61.4
Seq2	AAAACAAACCAACATTGGATT	59.6
Seq2kai	AGGAAGCTGGGTTGACATAGTC	63.2
Seq3	RTCAAATTGGATACAGAAAGA	54.6
Seq4	RGGTGACACAGCCTGGGA	56.0
Seq5	GGCCYCAGCCCACTGAGC	60.0
Seq6kai	ACATTACCACCGCTAAGATACAGAG	63.1
Seq7	TGCTYTTRACATCCTCACAGC	58.9
Seq8	GGCYGAACTGGAAGATGGAGC	68.2
Seq9	CAYCCAGGAGCGGGAAAGAC	67.1
Seq10	ACTAGAACCAAYGATTGGGA	56.0
Seq11	AGATGGYTGGATGCTAGGATC	60.5
Seq12	CATCCTGGACATAGATCTACG	58.6
Seq13	ACCATTGCRGTGTCAATGGC	67.0
Seq14	GCAGGACGAACRCTCAGAG	61.2
Seq15	ACTCCATTTGGACAACAGCG	65.0
Seq16	GATGGGACACAAGAATCACAC	61.9
Seq17	GAACTGATTGGCAGAGCCC	64.8
Seq18	CGGATTAAGCCATAGTACGG	60.9
T7	TAATACGACTCACTATAGGG	50.8
M13 Reverse	CAGGAAACAGCTATGAC	50.0
BGH reverse	TAGAAGGCACAGTCGAGG	59.0
DN-F	CTWTCAATATGCTGAAACGCG	63.2
DN-R	TCTATCCARAATYCCTGCTGTT	58.7
NanoLuc-185F	TCCCGTATGAAGGTCTGAGC	64.1
NanoLuc-291R	GCCATAGTGCAGGATCACCT	64.0
DV2-005-C-last-F	TATGCTGATTCCAACAGTGATGGCG	69.1
DV2-005-C-last-R	CGCCATCACTGTTGGAATCAGCATA	69.1
DV2-005-prM-last-F	GACAGCTGTCGCTCCTTCAATGACA	69
DV2-005-prM-last-R	TGTCATTGAAGGAGCGACAGCTGTC	69
DV2-026-C-last-F	TATGATGATCCCAACAGTGATGGCG	68.9
DV2-026-C-last-R	CGCCATCACTGTTGGGATCATCATA	68.9
DV2-E-371-395-F	ACATGGAAGGGAAAATCGTGCAACC	69.8
DV2-E-371-395-R	GGTTGCACGATTTTCCCTTCCATGT	69.8
DV2-E-641-665-F	TAGACCTGCCATTACCATGGCTGCC	70.7
DV2-E-641-665-R	GGCAGCCATGGTAATGGCAGGTCTA	70.7
DV2-E-936-956-F	GCAGAAACACAACATGGAAC	56.5
DV2-E-936-956-R	GTTCCATGTTGTGTTTCTGC	56.5
