# Supplementary material for: Genetic regions affecting the replication and pathogenicity of dengue virus type 2
Source: PLoS Negl Trop Dis. 2024 Jan 8;18(1):e0011885. doi: 10.1371/journal.pntd.0011885 (PMC10798627; doi:10.1371/journal.pntd.0011885)
Supplement: S2 Table — (PDF) [file pntd.0011885.s002.pdf]

S2 Table. Accession number list

A Asian-I type

Accession Number														
NC_001474.2	MW946586	MW946310	FJ744715	MW946279	MW946275	MW946404	GQ868624	EU482704	KY849767	MN448894	MN448928	MW946577	MN448734	MH827531
DQ181805	MW946549	MW946410	FJ744725	KF921930	MW946506	MW946285	MW946424	EU660414	KY849755	MW946602	MN448815	MN448769	MN448736	LC410188
ON398847	MW946490	MW946508	FJ639832	MW946500	MW946280	EU482542	MW946406	EU482703	KY849763	MN448920	MN448827	MN448816	MN448737	LC410187
DQ181804	MW946454	MW946487	FJ687438	MW946289	MW946378	EU482656	MW946305	FJ390385	KY849766	MN448929	MN448786	MN448834	MN448738	ON853797
DQ181803	MW946536	MW946440	FJ687437	MW946528	MW946569	EU482653	MW946319	EU482475	MW946367	MN448856	MN448813	MN448843	MN448749	MW788981
DQ181802	MW946553	MW946476	FJ744716	MW946588	MW946311	FJ873811	MW946302	FJ410202	MW946545	MN448887	MN448828	MN448817	MW946400	MW788982
OK469350	GQ868544	MW946509	FJ744714	MW946330	MW946396	EU482670	MW946597	EU482679	MN448865	MN448866	MN448790	MN448846	MW946544	ON890479
MW946393	MW946295	MW946432	MW946481	MW946407	MW946354	EU482673	MW946493	FJ205879	MN448923	MW946554	MN448767	MN448847	MN448771	OL414743
MW946474	MW946446	MW946452	MW946421	MW946418	MW946371	EU482644	MW946352	FJ410193	MN448904	MN448914	MN448783	MW946479	MW946266	OL414719
OK605759	MW946463	DQ181799	MW946538	MW946299	MW946309	EU482645	MW946355	FJ410224	MN448868	MW946510	MN448784	MN448839	MW946435	OL414721
MW946274	FJ906958	MW946514	FJ687441	MW946385	MW946353	EU482466	MW946272	EU482705	MN448879	MW946518	MN448791	MN448775	MW946550	OL414720
MW946515	MW946282	MW946445	FJ687442	MW946541	FM210214	EU569721	MW946459	EU687249	MN448895	MW946458	MN448803	MN448837	MW946304	OL414744
EU687246	MW946497	MW946283	FJ687443	MW946379	FM210216	EU482667	MW946533	FJ410228	MN448899	ON005157	MN448812	MN448844	MW946374	OL414761
MW946526	MW946361	MW946599	FJ687440	MW946262	EU482774	EU482665	MW946496	FJ461321	MN448897	MW946397	MN448819	MN448864	MW946431	OL414728
MW946260	MW946484	MW946503	FJ810409	MW946600	FM210215	EU482676	MW946322	FJ432726	MN448870	MW946585	MN448820	MN448776	MW946471	OL414729
MW946264	MW946298	MW946596	MW946438	MW946535	FM210240	EU482663	MW946336	EU660413	MN448882	KU509273	MN448822	MW946326	MW946286	MN923115
MW946356	MW946419	MW946368	MW946373	MW946335	FM210231	EU482470	JF730049	FJ410288	MW946525	MN448901	MN448842	MN448854	MN448766	ON908230
EU726767	GQ868545	MW946501	FJ687439	MW946542	FM210242	EU482543	EU482701	FJ410221	MW946531	MW946601	MN448823	MN448825	MN448762	MN923122
MW946425	MW946338	MW946261	MW946293	MW946524	EU482472	EU482450	FJ461305	GU131899	MW946568	MN448922	MN448789	MN448835	MN448772	MN923114
MW946539	MW946402	MW946565	MW946547	MW946488	FM210233	EU482652	EU482474	GQ868638	MN448838	MN448911	MN448793	MW946415	MN448763	MN923113
MW946394	MW946482	MW946473	FJ744713	MW946492	EU482775	EU482654	EU482702	GU131930	MN448867	MW946376	MN448781	MW946375	MN448764	MN923120
MW946412	MW946258	DQ181798	FJ810411	MW946529	FJ639708	EU482650	EU482700	JF730048	MN448924	MN448906	MN448787	MN448732	MN448797	MT754366
MW946346	MW946480	MW946325	FJ810412	GQ868621	FJ639709	EU482473	EU482698	JF730047	MN448861	MN448884	MW946382	MN448700	MN448761	MT754372
MW946450	MW946571	MW946332	FJ744710	FJ639705	GQ868623	EU482467	EU482699	GU131924	MN448873	MN448889	MN448859	MN448707	MN448773	MW788985
MW946328	MW946455	MW946306	FJ744712	GQ868622	FJ639710	EU482671	FJ562098	GQ868631	MW946486	MN448910	MN448927	MN448706	MN448774	MW788986
GQ868542	MW946598	MW946532	GU131886	GQ868620	FJ639711	EU482465	FJ410217	FJ639718	MN448908	MN448912	MN448778	MN448718	MN448785	MW788987
MW946422	MW946278	MW946276	FJ744711	FJ639704	MW946259	EU482646	FJ432724	GQ868625	MN448891	MN448919	MN448926	MN448699	MN448765	MW788984
MW946369	MW946513	MW946348	FJ687447	MW946391	MW946413	EU482642	MN448850	GU131932	MN448892	MN448900	MW946265	MN448726	MN448848	MW788983
MW946387	MW946570	MW946579	MW946594	FJ898452	MW946342	EU482464	MN448857	GU131931	MN448913	MN448903	MN448814	MN448739	MN448849	LC410185
MW946263	MW946464	MW946548	MW946427	MW946398	MW946448	EU482643	MN448874	JF730046	MN448925	MN448845	MN448833	MN448711	MN448853	MZ636801
MW946469	MW946477	MW946530	MW946423	MW946414	MW946392	EU482658	FJ547067	GU131902	MN448697	MN448881	MN448798	MN448701	MN448852	OL414734
OK469351	MW946357	MW946409	MW946429	MW946521	MW946324	EU482659	FJ205880	GU131900	MN448871	MW946386	MN448801	MN448712	MN448862	OL414735
MW946512	MW946460	MW946537	MW946489	MW946349	MW946430	EU482448	FJ410219	GU131898	MN448896	MN448860	MN448800	MN448735	MN448830	M84727
MW946499	MW946551	MW946277	MW946574	MW946563	MW946297	EU482447	EU677148	GU131901	MN448872	MN448890	MN448840	MN448742	MN448836	U87411
GQ868543	MW946592	MW946337	DQ181797	MW946358	MW946587	EU482446	EU677137	KF955402	MN448869	MN448888	MN448855	MN448728	MN448829	KU725663
MW946365	MW946257	MW946457	MW946578	MW946271	MW946340	EU482678	FJ410223	GU131928	MN448915	MN448824	MN448893	MN448720	MN448863	LC129169
DQ181800	MW946344	MW946296	MW946498	MW946395	MW946603	EU482445	EU660416	JF730045	MW946377	MW946567	MN448902	MN448733	KY672949	JQ045675
MW946593	MW946317	MW946426	MW946403	MW946441	MW946255	EU482675	FJ205877	GU131929	MN448880	MN448725	MN448875	MN448745	MF459663	JQ045676
MW946485	MW946290	MW946470	MW946561	MW946468	MW946517	EU482541	EU726776	KF955401	MW946442	MN448909	MN448876	MN448743	KY672947	JQ045672
MW946505	MW946350	MW946312	MW946453	MW946466	MW946595	EU482469	EU687250	KY849759	MW946557	MW946491	MN448821	MN448744	KY672948	JQ045671
MW946327	MW946399	MW946401	FJ687435	MW946347	FM210206	EU482471	EU660417	KY849752	MW946366	MN448858	MW946555	MN448698	MW946351	JQ045679
MW946591	MW946522	MW946362	FJ687436	MW946534	EU482777	EU482641	EU687248	FJ461309	MN448782	MN448916	MW946334	MN448704	MW946573	JQ045678
MW946511	MW946331	MW946294	FJ687434	MW946494	FM210207	EU482668	EU677149	FJ410241	MN448811	MW946300	MN448752	MN448708	MW946576	JQ045686
MW946284	MW946384	MW946303	FJ639828	MW946389	FM210205	EU482661	FJ410195	FJ410237	MN448832	MW946437	MN448755	MN448719	MW946323	JQ045685
MW946562	MW946417	MW946451	FJ744722	MW946273	FM210245	EU482677	FJ390387	FJ410233	MN448805	MN448780	MN448741	MN448723	MW946339	JQ045684
MW946372	MW946439	MW946447	FJ744721	MW946472	FM210246	EU482451	FJ547064	FJ461311	MN448779	MN448809	MN448746	MN448724	MW946405	JQ045683
MW946390	MW946590	MW946502	FJ744723	MW946388	EU482776	EU482647	EU482697	FJ410259	MN448804	MN448878	MN448748	MN448727	MW946589	JQ045680
MW946343	MW946315	MW946301	FJ744719	MW946416	MW946291	EU482655	FJ390384	FJ410215	MN448921	MN448917	MN448753	MN448729	MW946314	JQ045669
MW946287	MW946467	MW946307	FJ744720	MW946360	MW946316	EU482651	FJ205878	KY849757	MN448788	MN448918	MN448754	MN448730	MW946566	JQ045670
MW946408	MW946443	MW946516	FJ639830	MW946558	MW946318	EU482649	FJ410200	KY849765	MN448806	MN448885	MN448768	MN448731	MN018362	JQ045682
MW946363	MW946465	MW946288	FJ639829	MW946281	MW946320	EU482669	FJ410208	KY849756	MN448810	MN448770	MN448747	MN448740	MW946359	JQ045681
MW946383	MW946581	MW946267	FJ687444	EU482786	MW946552	EU482463	FJ024452	KY849760	MN448796	MN448795	MN448760	MN448705	MH888331	JQ045677
MW946256	MW946546	MW946523	FJ810410	FM210211	MW946268	EU482674	FJ024458	KY849753	MN448841	MN448799	MN448750	MN448709	MW946449	JQ045673
MW946556	MW946540	MW946434	FJ687446	EU482782	MW946321	EU482468	FJ024461	KY849754	MW946519	MN448794	MN448759	MN448710	MN018345	JQ045674
MW946572	MW946604	MW946341	FJ687445	FJ639707	MW946559	EU482657	EU660415	KY849764	MN448851	MN448802	MN448756	MN448714	KY672955	
MW946411	MW946504	MW946582	MW946520	FJ639706	MW946292	GU131896	FJ461314	KY849758	MN448702	MN448807	MN448757	MN448715	KY672945	
MW946420	MW946308	MW946436	MW946483	MW946580	MW946462	FJ639717	FJ859028	MN448877	MN448713	MN448808	MN448758	MN448716	KY672946	
FJ906957	MW946507	FJ744724	FJ639831	MW946380	MW946527	JN368476	FJ024454	MN448905	MN448703	MN448818	MN448777	MN448717	LC410186	
MW946364	MW946560	FJ744718	MW946428	MW946381	MW946270	GU131927	EU677138	MN448907	MN448883	MN448826	MN448751	MN448721	LC410184	
MW946543	MW946333	FJ744717	MW946456	MW946329	MW946370	GU131897	FJ373299	KY849768	MN448886	MN448831	MN448792	MN448722	MH827532	

S2 Table. Accession number list

B Cosmopolitan type

Accession Number												
MW946478	MW512366	MW512383	MH048672	MF156244	MK629886	MW512457	MH110601	MK858112	MK543449	MT006149	MW720947	OL414751
GQ398258	MW512367	MW512384	MH048673	MF156245	MF043956	MW512458	MH110602	MK858113	MK543450	MT006163	MW720948	OL414752
GQ398259	MW512368	MW512385	MH048675	MF156246	MK564477	MW512459	MH110603	MK858114	MK543471	MT006164	MW720949	OL414753
GQ398260	MW512369	MW512386	MH488959	MF156247	MK564478	MW512460	MH827525	MK858115	MK543479	MT006165	MW720950	OL414754
OK469352	MH822939	MW512387	MT832053	MF156248	MK564479	MW512461	MH827526	MK858116	MK564484	MT006184	MW720951	OL414755
JQ922549	MH822944	MW512388	MT832054	MF940236	MK564480	MW512462	MH827527	MN253134	MK564485	MT006185	MW720952	OL414756
DQ448231	MH822945	MW512389	MT832059	MF940237	MK578531	MW512463	MH827528	MZ312931	MK564486	MT006186	MW720953	OL414757
GQ252676	MH822946	MW512390	MT832060	MF940238	MK578532	MW512464	MH827529	MW730814	MK564487	MN328061	MW720954	OL414758
MW512341	MH822947	MW512391	MT832065	MF940239	MN018340	MW512465	MH827530	MW730815	MK564488	OL412740	MW720955	OL414764
GQ252677	MH822948	MW512392	MT832066	MF940240	MN018346	MW512466	MH827533	MW730816	MK578533	OL414717	MW720956	OL414765
FJ898454	MW512370	MW512393	MW512410	MF940241	MN018347	MW512467	MH827534	MW730817	MK783190	OL414718	MW720957	OL435143
KU509271	MW512371	MW512394	MW512411	MF940242	MN018348	LC410189	MH827535	MW730818	MK783191	OL414722	MZ453006	MW295816
MW945435	MW512372	MW512395	MW512412	MF940243	MN018350	LC410190	MH827536	MW730819	MK783192	OL414723	MZ453008	MW295818
MW512342	MW512373	MW512396	MW512413	MF940244	MN018353	MH010629	MH827537	MW730820	MK783193	OL414724	MZ453009	MW345921
MW512343	MW512374	MW512397	MW512414	MF940245	MN018358	MH110564	MH827539	MW730821	MK783194	OL414725	MZ453010	ON123649
MW512344	MW512375	MW512398	MW512415	MF940246	MN018363	MH110565	MH827540	MW730822	MK783195	OL414726	MZ453011	ON123651
MW512345	MW512376	MW512399	MW512416	MF940247	MN018364	MH110566	MH827541	MW730823	MK783196	OL414727	OK559627	ON123652
MW512346	MW512377	MW512400	MW512417	MF940248	MG592698	MH110567	MH827542	MW730824	MK783197	OL414730	OM368351	MW881533
MW512347	MW512378	MW512401	MW512418	MF940249	MG721054	MH110568	MH827543	MW730825	MK783198	OL414737	ON888666	OM639979
MW512348	MW512379	MW512402	MW512419	MF940250	MG721055	MH110569	MH827544	MW730826	MK783199	OL414738	ON890422	OM639980
MW512349	MW512380	MW512403	MW512420	MF940251	MG721056	MH110570	MH827545	MW730827	MK783201	OL414739	ON908221	OM639981
MW946433	MH822941	MW512404	MW512421	MF940252	MG721057	MH110571	MH827546	MW730828	MK783202	OL414740	ON908223	OM639982
MW946584	MH822950	MW512405	MW512422	MF940253	MG721058	MH110572	MH827547	MW730829	MK783203	OL414741	ON908224	OM639983
KF041236	MH822951	MW512406	MW512423	MN018337	MG721062	MH110573	MH827548	MW730830	MK783204	OL414742	ON908225	OM639984
MW512350	MH822956	MW512407	MW512424	MN018338	MH891768	MH110574	MH827549	MW730831	MK783205	OL414745	ON908227	OM639985
MW512351	MK858097	MW512408	MW512425	MN018349	MK858098	MH110575	MH827550	MW730832	MW721459	OL414747	ON908229	OM639986
MW512352	KJ010186	MW512409	MW512426	MN018351	MK858099	MH110576	MH827551	MW730833	MW721460	OL414759	ON908244	OM639987
MW512353	KJ701507	MN018339	MW512427	MN018352	MK858100	MH110577	MH827552	MW730834	MW721461	OL414760	ON885253	OM639988
MW387614	KM217158	MN018356	MW512428	MN018365	MK858101	MH110578	MH827553	MW730835	OM368352	OL414762	ON907581	OM639989
KF041235	MT832055	MN018357	MW512429	MN952966	MK858102	MH110579	MH827554	MW730836	ON875316	OL414763	MW186239	OM639990
KF041237	MT832056	MW721462	MW512430	MN952967	MK858103	MH110580	MK564476	MW730837	ON887640	OL420733	MW186240	OM639991
MW512354	MT832061	MW721463	MW512431	MH822953	MK858104	MH110581	MK564481	MW730838	ON908222	ON887284	MW512490	OM639992
MW512355	MT832062	MW721464	MW512432	MH822954	MK858105	MH110582	MK564482	MW730839	ON908226	MN923107	MW512491	OM639993
MW512356	MT832063	MW721465	MW512433	MT832057	MK858106	MH110583	MK564483	MW512468	MH891770	MN923108	MW512492	OM648092
MW721474	MT832064	MW721466	MW512434	MT832058	MK858107	MH110584	MK783189	MW512469	ON123663	MN923109	MW512493	OM680963
MW721475	MT832067	MW721467	KY672950	MW512435	MK858108	MH110585	MK783200	MW512470	ON799267	MN923110	MW512494	OM681318
KF360005	MT832068	MW721468	KY672951	MW512436	MK858109	MH110586	MN018341	MW512471	MW512479	MN923111	MW512495	OM698821
MW512357	MT832069	MW721471	KY672952	MW512437	MK858110	MH110587	MN018342	MW512472	MW512480	MN923112	MW512496	OM700180
MW512358	MT832070	MW721472	KY672953	MW512438	MK858111	MH110588	MN018343	MW512473	MW512481	MN923116	MW512497	OM700181
MW512359	MT832071	MW721473	KY672954	MW512439	MK411558	MH110589	MN018344	MW512474	MW512482	MN923117	MW512498	OM730078
MW512360	MT832072	MG560143	MF156233	MW512440	MK411559	MH110590	MN018354	MW512475	MW512483	MN923118	MZ636802	ON109598
MW512361	MT832073	MG560144	MF156234	MW512441	MN294937	MH110591	MN018355	MW512476	MW512484	MN923119	MZ636803	AF276619
KY427085	MT832074	MH822940	MF156235	MW512442	MF314189	MH110592	MN018359	MW512477	MW512485	MN923121	MZ636804	EU359009
KF041233	MT832075	MH822942	MF156236	MW512443	MW512449	MH110593	MN018360	MW512478	MW512486	MN944002	MZ636805	KY882458
KJ010185	MT832076	MH822943	MF156237	MW512444	MW512450	MH110594	MN018361	MN577545	MW512487	MT754367	OL414731	AB189122
KM217156	MT832077	MH822949	MF156238	MW512445	MW512451	MH110595	MW721469	MN577546	MW512488	MT754368	OL414732	AB189123
KM217157	MT832078	MH822952	MF156239	MW512446	MW512452	MH110596	MW721470	MN577547	MW512489	MT754369	OL414733	AB189124
MW512362	MT832079	MH822955	MF156240	MW512447	MW512453	MH110597	ON890351	MN577548	MT006142	MT754370	OL414736	AY858035
MW512363	MT832080	MK858096	MF156241	MW512448	MW512454	MH110598	ON907579	MN577549	MT006143	MT754371	OL414746	AY858036
MW512364	MW512381	MH823208	MF156242	MK629884	MW512455	MH110599	ON908228	LC410191	MT006144	MW720945	OL414749	AY776328
MW512365	MW512382	MH048671	MF156243	MK629885	MW512456	MH110600	MH891772	MK543448	MT006145	MW720946	OL414750	
