# Supplementary figures and images for: Genetic regions affecting the replication and pathogenicity of dengue virus type 2
Source: PLoS Negl Trop Dis. 2024 Jan 8;18(1):e0011885. doi: 10.1371/journal.pntd.0011885 (PMC10798627; doi:10.1371/journal.pntd.0011885)

S1 Figure

A

Viral RNA of transfection sup

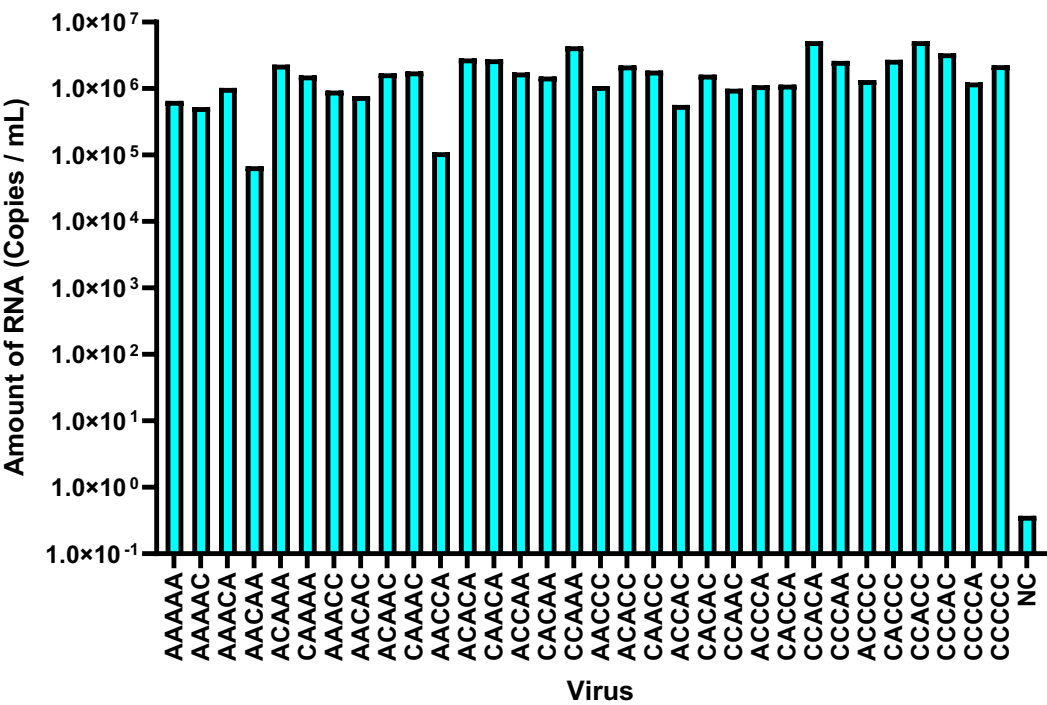

B

Viral RNA of propagation sup

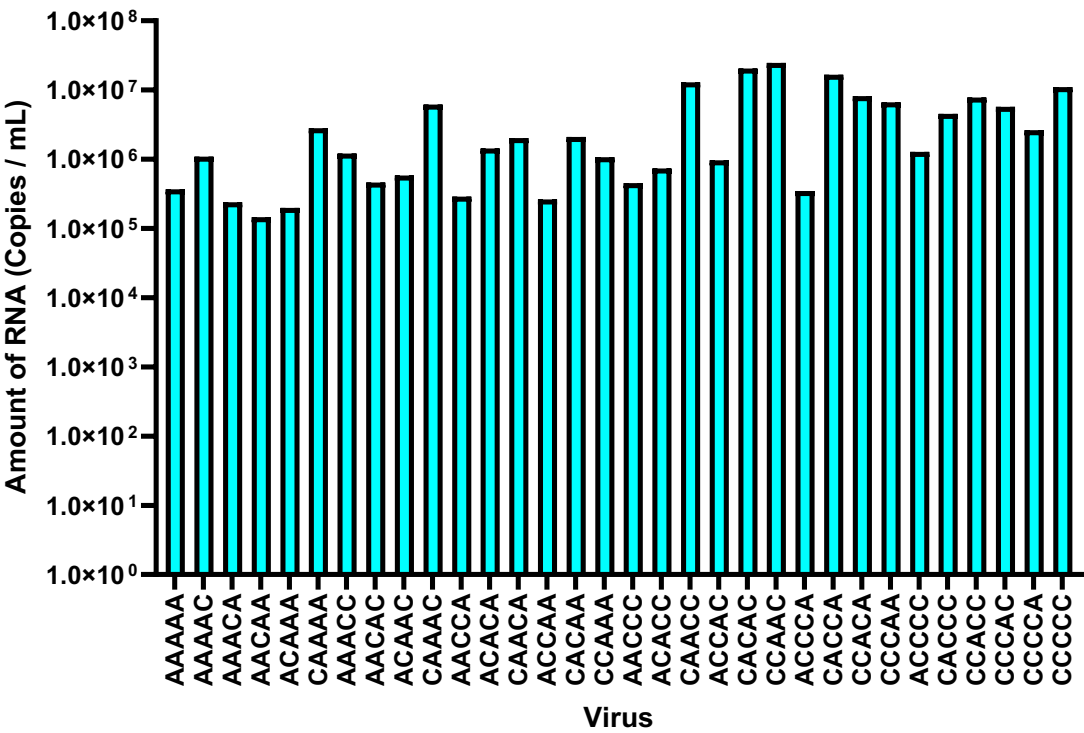

Supplement: S1 Fig — (A) Levels of viral RNA in the cell supernatants after the transfection of CPER products. (B) Levels of viral RNA in the cell supernatants after viral infection with the supernatants of the transfected cells. (PDF) [file pntd.0011885.s003.pdf]

S2 Figure

A

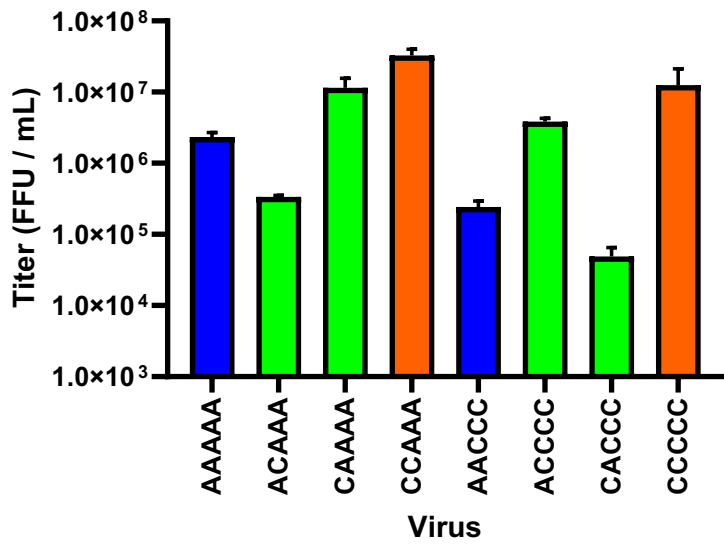

B

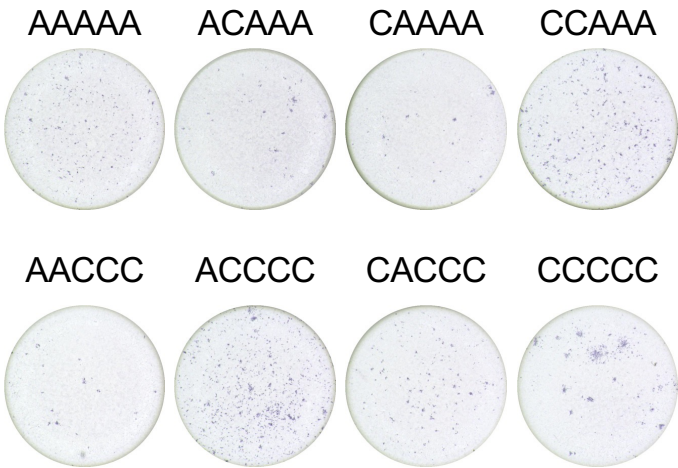

Supplement: S2 Fig — (A) Infectious titers of an independent lot of stock viruses. (B) Focus-forming unit of an independent lot of stock viruses 3 days after the infection of Vero cells at a multiplicity of infection of 0.5 copies/cell. The number of seeding Vero cells was 4.0 × 10^4^ cells/well. These experiments were performed using the method described in the "Focus-forming assay" section of the Materials and Methods. (PDF) [file pntd.0011885.s004.pdf]
