# Supplementary material for: Genetic regions affecting the replication and pathogenicity of dengue virus type 2
Source: PLoS Negl Trop Dis. 2024 Jan 8;18(1):e0011885. doi: 10.1371/journal.pntd.0011885 (PMC10798627; doi:10.1371/journal.pntd.0011885)
Supplement: S3 Fig — (A, B, C) Results of three independent experiments on Replicon assay. The fold increases in luciferase activity when compared to the activity at 24 h after the transfection of DENV2 replicons are shown as the means and standard deviation (SD) of triplicate samples. Statistical significance of the differences were tested using the Holm-Sidak method with alpha = 0.05. Each row was analyzed individually without assuming a consistent SD. (PDF) [file pntd.0011885.s005.pdf]

S3 Figure

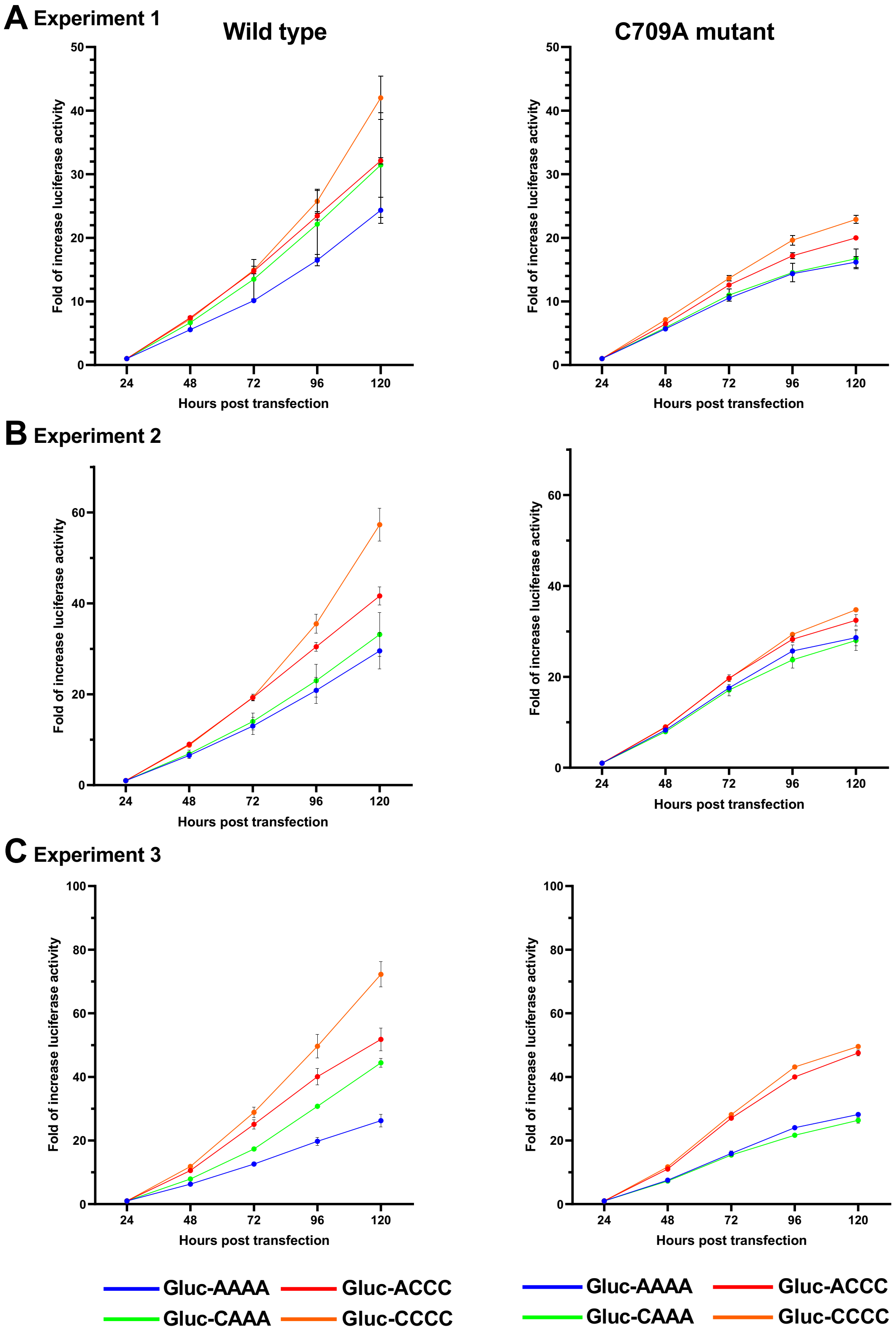
A Experiment 1
Wild type
C709A mutant
B Experiment 2
C Experiment 3
Fold of increase luciferase activity
Hours post transfection
24
48
72
96
120
Gluc-AAAA
Gluc-ACCC
Gluc-CAAA
Gluc-CCCC
