# Supplementary material for: Genetic regions affecting the replication and pathogenicity of dengue virus type 2
Source: PLoS Negl Trop Dis. 2024 Jan 8;18(1):e0011885. doi: 10.1371/journal.pntd.0011885 (PMC10798627; doi:10.1371/journal.pntd.0011885)
Supplement: S4 Fig — (A) Body weight changes of the mice shown in Fig 8A and 8B. (B) Body weight changes of the mice shown in Fig 8C and 8D. (C) Body weight changes of the mice shown in Fig 8E and 8F. In all data, “-” indicates after euthanization. Parts with no data are left blank. (PDF) [file pntd.0011885.s006.pdf]

S4 Figure

A

Virus	Copies/body	Days after infection													
		0	1	2	3	4	5	6	7	8	9	10	11	12	13
AAAAA	5.0E+05	16.87	17.75	18.17	19.71	19.60	20.08	20.15	19.27	20.95	20.86	21.46	21.94	22.33	22.30
		13.98	16.03	16.60	17.28	17.56	17.14	16.64	16.90	19.09	20.08	19.88	20.59	20.74	20.15
		11.32	12.26	12.15	13.48	14.04	13.94	13.42	13.10	14.24	15.33	15.67	15.58	15.98	15.97
	5.0E+04	11.28	10.06	11.00	11.78	12.54	12.48	11.33	10.10	9.98	9.43	-	-	-	-
		14.25	14.76	15.08	14.96	15.70	15.66	15.15	15.09	15.72	16.26	16.27	16.40	16.32	16.71
		13.41	14.60	14.60	14.97	15.50	15.77	14.97	14.69	15.98	16.92	16.65	16.61	16.67	16.66
	5.0E+03	10.94	12.09	12.35	13.50	13.02	13.90	13.65	12.81	12.86	13.92	15.07	15.41	16.05	16.68
		14.90	15.74	16.48	17.34	17.81	18.36	19.00	18.65	18.85	19.93	20.36	20.38	20.70	20.92
		11.55	12.67	12.76	14.12	14.35	15.25	15.51	15.48	16.14	17.55	18.23	18.44	18.50	19.92
CCCCC	5.0E+05	13.28	14.26	14.92	15.42	15.77	13.13	12.45	-	-	-	-	-	-	-
		15.24	15.92	16.51	16.55	16.81	14.06	13.21	-	-	-	-	-	-	-
		14.42	14.73	14.87	15.33	15.41	12.75	12.02	-	-	-	-	-	-	-
	5.0E+04	12.57	12.72	13.11	13.81	13.75	12.78	11.20	10.26	-	-	-	-	-	-
		13.99	14.63	15.17	16.07	16.21	16.24	13.78	12.28	11.89	-	-	-	-	-
		13.33	13.60	14.24	15.28	14.87	13.54	12.13	11.47	-	-	-	-	-	-
	5.0E+03	16.03	16.97	17.67	18.36	18.75	18.39	15.78	14.61	-	-	-	-	-	-
		10.13	10.46	10.86	11.83	11.34	12.40	12.80	11.66	11.21	11.42	-	-	-	-
		12.44	12.54	12.85	14.99	15.72	16.22	14.56	12.59	12.11	-	-	-	-	-

B

Virus	Days after infection													
	0	1	2	3	4	5	6	7	8	9	10	11	12	13
AAAAA	18.33	18.18	18.78	19.14	19.06	18.50	18.15	18.22	19.41	19.44	20.34	19.94	20.37	20.48
	18.25	18.24	18.16	18.22	18.49	18.11	17.21	17.23	17.89	18.00	18.64	19.45	19.63	19.35
	17.80	18.24	17.50	17.29	17.16	17.08	16.68	17.53	17.16	17.48	17.17	17.50	17.53	17.98
	10.30	10.55	11.30	11.79	12.12	11.57	10.93	10.91	12.40	12.88	13.21	13.98	14.51	14.86
	11.58	12.27	12.83	13.10	13.39	12.78	12.11	11.77	13.17	14.28	14.97	15.27	15.50	15.91
CCCCC	18.41	18.04	18.35	18.10	17.18	14.97	14.39	-	-	-	-	-	-	-
	20.15	20.01	20.21	20.33	19.33	16.87	16.14	15.36	-	-	-	-	-	-
	21.74	21.48	21.26	20.80	20.11	17.73	17.21	-	-	-	-	-	-	-
	8.30	8.88	9.51	9.32	8.80	-	-	-	-	-	-	-	-	-
	11.18	11.57	12.17	12.53	12.54	11.29	10.53	9.89	-	-	-	-	-	-
CAAAA	17.19	17.63	17.25	17.08	16.96	15.74	14.88	14.30	14.93	16.00	16.18	16.49	16.16	16.07
	17.85	16.83	16.82	16.42	16.53	15.84	14.81	14.68	16.39	16.43	16.47	16.82	16.59	16.28
	17.40	17.39	17.53	17.39	17.37	16.70	15.87	15.16	16.57	16.96	16.82	16.93	16.42	16.35
	11.20	12.15	13.23	13.66	14.30	12.24	11.85	11.58	-	-	-	-	-	-
	12.00	13.23	14.07	14.71	15.60	14.04	12.84	-	-	-	-	-	-	-
ACCCC	20.96	20.98	20.53	20.58	20.67	19.23	18.48	18.04	18.96	20.01	20.17	20.83	21.19	21.23
	20.93	21.30	21.61	21.00	21.31	19.95	19.07	18.20	19.17	20.43	20.78	21.45	21.85	22.33
	8.66	9.52	9.34	10.16	10.00	8.72	8.10	-	-	-	-	-	-	-
	12.35	13.14	13.29	13.73	13.85	12.08	11.33	10.53	-	-	-	-	-	-
	17.80	18.09	17.71	17.90	17.72	16.24	16.07	15.65	17.01	18.09	18.18	18.35	18.31	18.64
CCAAA	17.15	17.13	16.85	16.56	15.55	13.67	13.03	12.30	-	-	-	-	-	-
	16.15	16.41	16.54	16.14	14.66	13.12	12.44	11.80	-	-	-	-	-	-
	18.21	17.53	17.63	17.27	16.69	14.62	13.90	13.23	-	-	-	-	-	-
	13.55	14.72	15.47	13.95	12.01	-	-	-	-	-	-	-	-	-
AACCC	18.86	18.04	18.43	18.19	18.56	18.65	18.11	18.39	19.08	18.70	19.47	19.41	19.57	20.04
	17.90	17.84	17.53	17.49	17.11	17.12	16.19	15.53	16.44	16.96	17.43	17.51	17.53	17.49
	19.17	18.55	19.10	18.43	18.16	18.20	17.97	18.08	18.60	18.49	18.91	19.06	19.04	19.33
	11.76	12.37	13.61	14.12	14.54	14.30	14.00	13.63	14.80	16.04	16.39	16.38	16.72	16.81
	20.63	20.88	20.97	20.73	21.13	21.60	22.13	21.65	21.99	22.80	22.43	22.24	22.31	22.70

S4 Figure

C

Virus	Days after infection													
	0	1	2	3	4	5	6	7	8	9	10	11	12	13
AAAAA	11.35	12.04	11.78	12.86	12.80	13.00	12.06	11.86	13.74		14.15	15.29	15.40	15.79
	13.38	14.02	14.05	14.79	14.84	15.33	14.44	14.55	16.24		16.38	17.15	16.77	17.12
	10.55	11.56	11.91	12.66	12.50	12.27	11.15	11.08	11.61		12.64	13.55	13.04	12.37
CCCCC	17.40	17.76	18.15	18.91	18.11	15.58	14.37	13.90	-	-	-	-	-	-
	15.31	16.43	16.86	17.48	16.61	14.07	13.26	12.81	-	-	-	-	-	-
	10.64	11.41	11.35	11.68	11.09	10.02	9.49	-	-	-	-	-	-	-
	12.73	13.96	14.91	15.10	14.62	12.43	11.20	-	-	-	-	-	-	-
	13.38	13.16	14.77	15.39	15.58	15.41	13.36	12.56	-	-	-	-	-	-
ACAAA	11.35	11.13	12.39	13.63	13.40	13.44	11.91	11.36	10.68		-	-	-	-
	11.76	12.28	12.96	13.91	13.76	11.75	10.81	10.35	-	-	-	-	-	-
	11.14	11.38	11.54	12.66	12.44	11.22	9.97	9.45	-	-	-	-	-	-
	12.81	13.44	14.01	15.42	15.39	15.16	12.69	12.02	12.00		10.71	10.06	-	-
	11.52	12.32	13.11	14.14	14.19	12.78	11.45	10.90	-	-	-	-	-	-
CACCC	9.18	10.08	10.45	10.78	11.03	10.16	9.01	-	-	-	-	-	-	-
	13.31	13.85	13.99	15.52	14.89	14.96	13.93	13.96	15.01		15.69	16.18	16.40	16.17
	12.47	13.21	13.59	15.06	14.84	13.94	12.68	12.26	12.56		9.66	-	-	-
	14.41	15.20	15.46	17.01	16.45	16.02	14.65	13.69	14.15		15.19	15.22	14.59	13.25
	12.69	13.81	14.88	15.90	16.10	14.80	13.67	13.07	13.22		-	-	-	-
