# Supplementary material for: Genetic regions affecting the replication and pathogenicity of dengue virus type 2
Source: PLoS Negl Trop Dis. 2024 Jan 8;18(1):e0011885. doi: 10.1371/journal.pntd.0011885 (PMC10798627; doi:10.1371/journal.pntd.0011885)
Supplement: S5 Fig — Differences in amino acid residues in two structural proteins and three non-structural proteins between the Asian-I and Cosmopolitan types. Amino acid residues found in Th16-005DV2 and Th16-026DV2 are shown in bold font. For each position, the amino acid residue and its frequency (percentage) in the sequences are shown. The number of sequences used was 894 for the Asian-I type, and 662 for the Cosmopolitan type (S2 Table). (PDF) [file pntd.0011885.s007.pdf]

S5 Figure

Genotype	prM-15			prM-16			prM-29			prM-52			prM-82			prM-127			prM-148		
	aa	Count	%	aa	Count	%	aa	Count	%	aa	Count	%	aa	Count	%	aa	Count	%	aa	Count	%
Asian-I (n=894)	**G**	**725**	**81.1**	**I**	**850**	**95.1**	**D**	**894**	**100**	**K**	**894**	**100**	**T**	**889**	**99.4**	**I**	**892**	**99.8**	**H**	**891**	**99.7**
	S	168	18.8	M	27	3.0							I	3	0.3	V	2	0.2	Y	3	0.3
	X	1	0.1	R	17	1.9							A	2	0.2						
Cosmopolitan (n=662)	**S**	**661**	**99.8**	**R**	**644**	**97.3**	**N**	**488**	**73.7**	**N**	**662**	**100**	**A**	**655**	**98.9**	**V**	**456**	**68.9**	**Y**	**588**	**88.8**
	-	1	0.2	K	16	2.4	D	169	25.5				M	4	0.6	I	199	30.1	H	68	10.3
				X	1	0.2	E	2	0.3				T	2	0.3	L	4	0.6	N	3	0.5
				-	1	0.2	X	2	0.3				V	1	0.2	A	3	0.5	X	2	0.3
							-	1	0.2										C	1	0.2

Genotype	Env-52			Env-71			Env-83			Env-141			Env-149			Env-226			Env-228			Env-346			Env-390			Env-462			Env-484		
	aa	Count	%	aa	Count	%	aa	Count	%	aa	Count	%	aa	Count	%	aa	Count	%	aa	Count	%	aa	Count	%	aa	Count	%	aa	Count	%	aa	Count	%
Asian-I (n=894)	**Q**	**892**	**99.8**	**E**	**894**	**100**	**K**	**887**	**99.2**	**V**	**889**	**99.4**	**H**	**889**	**99.4**	**K**	**734**	**82.1**	**E**	**670**	**74.9**	**Y**	**887**	**99.2**	**N**	**893**	**99.9**	**I**	**893**	**99.9**	**I**	**868**	**97.1**
	R	1	0.1				N	7	0.8	I	4	0.4	R	3	0.3	T	157	17.6	G	224	25.1	H	7	0.8	S	1	0.1	M	1	0.1	V	23	2.6
	L	1	0.1							A	1	0.1	Y	1	0.1	R	2	0.2													T	3	0.3
													Q	1	0.1	E	1	0.1															
Cosmopolitan (n=662)	**H**	**478**	**72.2**	**A**	**659**	**99.5**	**N**	**653**	**98.6**	**I**	**535**	**80.8**	**N**	**655**	**98.9**	**T**	**598**	**90.3**	**G**	**662**	**100**	**H**	**651**	**98.3**	**S**	**654**	**98.8**	**V**	**662**	**100**	**V**	**603**	**91.1**
	Q	169	25.5	S	3	0.5	K	5	0.8	V	126	19.0	K	4	0.6	K	42	6.3				Y	6	0.9	N	8	1.2				I	57	8.6
	Y	9	1.4				S	4	0.6	X	1	0.2	X	2	0.3	I	17	2.6				Q	4	0.6							F	1	0.2
	L	6	0.9										S	1	0.2	R	4	0.6				L	1	0.2							X	1	0.2
																A	1	0.2															

Genotype	NS1-50			NS1-80			NS1-117			NS1-128			NS1-129			NS1-131			NS1-174			NS1-177			NS1-222			NS1-247			NS1-264			NS1-272			NS1-281			NS1-286		
	aa	Count	%	aa	Count	%	aa	Count	%	aa	Count	%	aa	Count	%	aa	Count	%	aa	Count	%	aa	Count	%	aa	Count	%	aa	Count	%	aa	Count	%	aa	Count	%	aa	Count	%	aa	Count	%
Asian-I (n=894)	**Q**	**888**	**99.3**	**A**	**888**	**99.3**	**T**	**893**	**99.9**	**S**	**888**	**99.3**	**Y**	**612**	**68.5**	**Q**	**893**	**99.9**	**K**	**874**	**97.8**	**A**	**878**	**98.2**	**N**	**893**	**99.9**	**L**	**894**	**100**	**I**	**867**	**97.0**	**K**	**893**	**99.9**	**D**	**869**	**97.2**	**I**	**673**	**75.3**
	H	4	0.4	S	4	0.4	X	1	0.1	P	5	0.6	H	278	31.1	K	1	0.1	R	20	2.2	V	9	1.0	S	1	0.1				T	19	2.1	R	1	0.1	E	22	2.5	V	192	21.5
	R	1	0.1	T	1	0.1				F	1	0.1	X	2	0.2							T	5	0.6							V	8	0.9				G	1	0.1	T	27	3.0
	K	1	0.1	V	1	0.1							F	1	0.1							I	2	0.2													V	1	0.1	L	1	0.1
													Q	1	0.1																						X	1	0.1	M	1	0.1
Cosmopolitan (n=662)	**H**	**652**	**98.5**	**T**	**359**	**54.2**	**A**	**460**	**69.5**	**L**	**489**	**73.9**	**H**	**654**	**98.8**	**H**	**489**	**73.9**	**R**	**632**	**95.5**	**V**	**616**	**93.1**	**S**	**656**	**99.1**	**F**	**647**	**97.7**	**T**	**662**	**100**	**R**	**440**	**66.5**	**E**	**659**	**99.5**	**V**	**660**	**99.7**
	Y	8	1.2	S	300	45.3	T	201	30.4	P	148	22.4	Y	7	1.1	Q	173	26.1	K	29	4.4	A	42	6.3	N	6	0.9	L	13	2.0				K	222	33.5	R	2	0.3	M	1	0.2
	Q	1	0.2	L	1	0.2	X	1	0.2	H	20	3.0	Q	1	0.2				X	1	0.2	X	2	0.3				I	1	0.2							X	1	0.2	X	1	0.2
	R	1	0.2	P	1	0.2				F	2	0.3										I	1	0.2				Y	1	0.2												
				X	1	0.2				X	2	0.3										T	1	0.2																		
										S	1	0.2																														

Genotype	NS2A-5			NS2A-51			NS2A-57			NS2A-65			NS2A-120			NS2A-158			NS2A-171			NS2A-215		
	aa	Count	%	aa	Count	%	aa	Count	%	aa	Count	%	aa	Count	%	aa	Count	%	aa	Count	%	aa	Count	%
Asian-I (n=894)	**V**	**834**	**93.3**	**K**	**888**	**99.3**	**V**	**875**	**97.9**	**A**	**632**	**70.7**	**V**	**641**	**71.7**	A	856	95.7	**I**	**891**	**99.7**	**N**	**873**	**97.7**
	I	59	6.6	R	5	0.6	M	18	2.0	T	248	27.7	I	251	28.1	**T**	**38**	**4.3**	V	2	0.2	S	21	2.3
	X	1	0.1	X	1	0.1	X	1	0.1	V	14	1.6	A	2	0.2				T	1	0.1			
Cosmopolitan (n=662)	**I**	**659**	**99.5**	**R**	**662**	**100**	**M**	**657**	**99.2**	**T**	**658**	**99.4**	**I**	**654**	**98.8**	**A**	**662**	**100**	**A**	**289**	**43.7**	**S**	**488**	**73.7**
	V	3	0.5				I	2	0.3	A	3	0.5	V	5	0.8				I	223	33.7	N	173	26.1
							L	1	0.2	X	1	0.2	M	3	0.5				T	145	21.9	D	1	0.2
							T	1	0.2										V	5	0.8			
							X	1	0.2															

Genotype	NS2B-21			NS2B-57			NS2B-59			NS2B-60			NS2B-63			NS2B-65			NS2B-112		
	aa	Count	%	aa	Count	%	aa	Count	%	aa	Count	%	aa	Count	%	aa	Count	%	aa	Count	%
Asian-I (n=894)	**L**	**892**	**99.8**	**T**	**870**	**97.3**	**V**	**894**	**100**	**K**	**893**	**99.9**	**D**	**881**	**98.5**	**A**	**892**	**99.8**	**I**	**655**	**73.3**
	F	2	0.2	A	23	2.6				R	1	0.1	E	11	1.2	P	1	0.1	V	227	25.4
				X	1	0.1							N	1	0.1	X	1	0.1	M	11	1.2
													X	1	0.1				T	1	0.1
Cosmopolitan (n=662)	L	379	57.3	**A**	**660**	**99.7**	V	373	56.3	**R**	**661**	**99.8**	**E**	**488**	**73.7**	A	660	99.7	**V**	**658**	**99.4**
	**F**	**283**	**42.7**	V	1	0.2	**I**	**288**	**43.5**	X	1	0.2	D	173	26.1	**V**	**1**	**0.2**	I	2	0.3
				X	1	0.2	X	1	0.2				X	1	0.2	X	1	0.2	L	1	0.2
																			M	1	0.2
