# Supplementary material for: Genetic regions affecting the replication and pathogenicity of dengue virus type 2
Source: PLoS Negl Trop Dis. 2024 Jan 8;18(1):e0011885. doi: 10.1371/journal.pntd.0011885 (PMC10798627; doi:10.1371/journal.pntd.0011885)
Supplement: Attachment — Submitted filename: Cover letter20230501-2.docx [file pntd.0011885.s008.docx]

Dear Editor of ***PLOS Neglected Tropical Diseases***,

Enclosed is a research article entitled “**Genetic regions affecting the replication and pathogenicity of dengue virus type 2**”, manuscript number **PNTD-D-23-00516**. This is a transfer from ***PLOS PATHOGENS*** and we have just changed the order of “**Results**” and “**Methods**” sections according to the Submission Guideline of ***PLOS Neglected Tropical Diseases.*** We would greatly appreciate your consideration for publication of this manuscript.

I am looking forward to your reply.

Very truly yours,

Tatsuo Shioda

Department of Viral Infections

Research Institute for Microbial Diseases

Osaka University

3-1, Yamada-oka, Suita

Osaka 565-0871, Japan

Tel. +81-6-6879-8346

E-mail: [shioda@biken.osaka-u.ac.jp](mailto:shioda@biken.osaka-u.ac.jp)
