# Supplementary material for: Genetic regions affecting the replication and pathogenicity of dengue virus type 2
Source: PLoS Negl Trop Dis. 2024 Jan 8;18(1):e0011885. doi: 10.1371/journal.pntd.0011885 (PMC10798627; doi:10.1371/journal.pntd.0011885)
Supplement: Attachment — Submitted filename: ResponseToReviewers2.docx [file pntd.0011885.s009.docx]

Our responses to the Editors’ and Reviewers’ comments are as follows;

**Editors:**

The reviewers raised multiple relevant points regarding your study that must be addressed for publication. First, all reviewers noted you did not include figure legends which is a major issue when reviewing the manuscript. Second, all reviewers questioned why virus stocks were not generated, sequenced, and used to compare viral growth. It will be critical to complete these experiments. Finally, reviewer 1 makes a good point regarding proper controls for all experiments. Given these major criticisms and those below, I recommend you address all of the reviewers points for resubmission.

**Responses**

1. We sincerely apologize for the lack of figure legends, which caused serious problems in interpreting our figures.
2. We had generated virus stocks. To clarify this point, we revised several portions of the manuscript. We also performed post-hoc sequence analysis of all virus stocks, and found that there was one unintended mutation in one chimera (Figure 4). Exclusion of this chimera did not affect our conclusions. This information has been added to the revised manuscript.
3. We appreciate Reviewer 1’s suggestion regarding the negative controls, and repeated the experiment accordingly. The results from the new experiment were added to the revised manuscript.

Our responses to the reviewers’ comments are as follows.

**Reviewer #1:**

**<Methods>**

The study aims at mapping the genetic determinants responsible for the differences in cell culture growth of the Cosmopolitan and Asian I genotypes of Dengue virus 2 (DENV2). To this end it follows a genetic approach based on the construction of chimeric viruses and the assessment of their phenotypes in vitro and in vivo. The genetic system relies on the production of virus RNA genomes from a CMV promoter in transfected cells. While the system appears versatile to generate the large number of chimeras used in the study, conclusions made from the generation virus genomes based on RNA transcription in the cell nucleus need to consider the limitations of the system.

**Response:** We appreciate this comment. In the revised manuscript, we added one paragraph to the Discussion section to describe the limitations of the CPER method (lines 441-449).

(1) How do the authors control for differences in transfection efficiencies? Throughout the manuscript, the comparisons of virus growth in culture should be assessed using virus stocks to perform growth curves as shown in Figure 1. This is specially necessary to compare Cosmopolitan and Asian I recombinant types in Figure 3, and combination of envelope fragments in Figure 9.

**Response:** First of all, we sincerely apologize for the lack of figure legends, which made it difficult to interpret the figures. We did not control for differences in transfection efficiencies, but we did generate virus stocks and measured the levels of viral RNA in the stocks with a real-time RT-PCR assay to normalize the input viruses for the experiments. To clarify this point in the manuscript, we added more detailed explanation to the Results section, and to Figures 3, 4, and 9 in lines 241-243, 255-256, and 404-405, respectively.

(2) Figure 2 and lines 226-227. Please describe what the negative control consists of. Mock DNA transfections may not be appropriate controls. Instead, a control construct bearing a a mutation in the NS5 RdRp catalytic site would be more meaningful.

**Response:** We appreciate this comment. As negative controls, we used NS5-C709A mutants in both the Cosmopolitan and Asian-I backgrounds. The mutant viruses did not grow at all, and the results are shown in Figure 2 and lines 228-240 of the revised manuscript.

(3) Figure 3 and lines 233-235. The rationale of the approach is not properly described in the text. In panel A, are foci size measured in cells infected with the supernatants of cells transfected with CPER products? If this is the case, were cells infected at the same MOI? In panel B, while quantification of RNA copies in the supernatant shows differences within one order of magnitude for the Cosmopolitan type compared to the Asian I type (left), differences in infectious virus production are at least >20-fold (right). This is also an indication of the impact of the genetic background on virus infectivity that is overlooked in the text. Finally, and referred to comment (1), curves of RNA or virus production as a function of time after transfection of CPER products are not appropriate to measure growth kinetics.

**Response:** We appreciate these valuable comments. As we described in the response to the first comment, we generated virus stocks, and measured the levels of viral RNA in the stocks to normalize the input viruses for the experiments. To clarify this point in the manuscript, we added more explanations in the Results section for Figures 3 at lines 241-243 of the revised manuscript. We also added a comment on the discrepancy between levels of viral RNA and infectivity in lines 245-249 of the revised manuscript.

(4) Figure 7. In the replicon assay using Gaussia luciferase as a reporter, lines 291-294 claim that luciferase levels measured in the supernatant of transfected cells as of 48 hours post-transfection reflect authentic genome replication. Characterization of the assay previously reported by the authors (DOI: 10.7883/yoken.67.209) showed that differences in luciferase levels for the wild type construct and an NS5 mutant control are < 10-fold. Therefore, it is arguable whether differences observed for the Cosmopolitan and Asian I types in the figure reflect functionality of the swapped NS1-2B fragment. The NS5 mutant construct needs to be included in the analysis.

**Response:** We appreciate this comment. According to your comment, we used a polymerase-inactivated mutant of each of the wild-type and chimeric viruses. Inclusion of these negative controls verified our conclusions, and this information was added to the revised manuscript (lines 317-318, and 328-336).

(5) Figure 8D and 8F. Lethality of the Asian I type is disparate between panels. While no lethality is registered in panel D, a 40% lethality is reported in panel F for the same virus. Since chimeric viruses result in intermediate lethality phenotypes, the differences between experiments for the same virus argue against the significance of conclusions.

**Response:** We sincerely apologize for our careless, but very serious, mistake in this figure. In both the previous and revised versions of panel C in S2 Fig., which showed the body weight changes of the mice in panel F, none of the mice actually succumbed to Asian-I infection. Their body weights ranged from 10.55 g to 13.38 g at the time of infection (S2 Fig.). When we performed this experiment, we also tested two much smaller mice (5.1 g and 6.8 g), and both of them succumbed to Asian-I infection. These two mice were outliers, but we included them in Figure 8F by mistake. In the revised manuscript, we removed the data of these two small mice from panel F of Figure 8. During the process of outlier detection, we realized that one mouse infected with CCAAA virus was similarly small (6.54 g). We therefore removed the data of this mouse from panel D of Figure 8 and S2 Fig. Removal of the data of this mouse did not affect any of the conclusions.

**<Results>**
Figure legends are not provided in the current version of the manuscript thus information regarding statistical analyses (number of replicate experiments, tests applied, significance of the observed differences) is not available. It is therefore not possible to judge the soundness of the data.

**Response:** We sincerely apologize for the lack of figure legends. In the revised manuscript, we provided figure legends that describe the number of replicate experiments, tests applied, and significance of the observed differences.

< **Conclusions** >
The limitations of the CPER method need to be clearly stated in the text as already commented above.

**Response:** As suggested, we added one paragraph to the Discussion section of the revised manuscript to describe the limitations of the CPER method (lines 441-449).

In addition, construction of homology models can serve as an approach to define likely interactions between residues. In these models whether two residues are interacting is based only in geometric criteria, i.e. the distance between residues in the model. In particular, for hydrogen bonding also the angles confine the possibility of interaction between residues. Therefore, assumptions made on the impact of residue differences between Cosmopolitan and Asian I types on envelope proteins maturation and dynamics (lines 417-424) should be toned down. Alternatively, molecular dynamics calculations can be performed to further support the proposed model.

**Response:** We appreciate this comment. We deleted our assumption on hydrogen bonds, and only pointed out the possibility of an ionic bond, and we also toned down the corresponding paragraph in the revised manuscript (lines 472-484).

< **Editorial and Data Presentation Modifications?**>

References are not uniformly formatted.

**Response:** We apologize for our careless mistakes in formatting the references. We have carefully reformatted the references in the revised manuscript.

<**Summary and General Comments**>

The manuscript by Samune and colleagues presents a systematic analysis of the viral determinants that account for the differences in the in vitro and in vivo fitness of the Cosmopolitan and Asian I genotypes of dengue virus serotype 2 (DENV2). The two genotypes of the virus co-circulate in Thailand together with viruses of the other three serotypes. The initial observation is that isolates of these two genotypes display differences in foci size in mammalian cells and growth kinetics in mosquito and mammalian cells, with the Cosmopolitan type showing bigger foci size and faster growth. Using a circular extension polymerase reaction (CPER) to construct a series of chimeric cDNAs of Cosmopolitan and Asian I types that launch full length RNA virus genomes from the CMV promoter the authors are able to narrow down the regions responsible for the observed differences to the structural proteins (C-prM-E) and non structural proteins (NS) 1, 2A, and 2B. Further dissection of the structural proteins coding region indicates that chimeric viruses bearing Cosmopolitan type prM and E proteins in the Asian I type background yield higher virus titers and form bigger foci compared to the parental Asian I type. The data also point to a necessary association between prM and E proteins that is also evidenced using a reporter replicon trans packaging system: replicon RNA is packaged into particles consisting of chimeras of the prM and E proteins, but reporter gene activity is only detected in cells infected with the assembled particles when particles carry prM and E of the homologous type. In turn, the CPER approach is also used to construct cDNA replicons carrying Gaussia luciferase in place of virus structural proteins to address the contribution of NS1, NS2A, and NS2B to the phenotype of Cosmopolitan and Asian I types. Results indicate that insertion of Asian I typr NS1-2B fragment in the Cosmopolitan type background results in impaired luciferase activity compared to the parental construct, while insertion of the Cosmopolitan type fragment in the Asian I type background enhances luciferase activity. In experimental infections of immunodeficient mice, the Cosmopolitan type virus reaches higher titers than the Asian I type and results in increased lethality. In this model, chimeric viruses bearing heterologous structural or NS1-2B fragments display phenotypes between the parental Asian I and Cosmopolitan types. Finally, the determinants responsible for the differences in the cell culture phenotype are pinpointed to five residues in the E coding sequence that differ between the two types. Mapping of residues into molecular models suggests an involvement of these residues in the interaction between E dimers on the virion surface and in the contact between E and prM.

Overall, the manuscript provides a descriptive analysis of virus phenotypes that follows a systematic characterization of chimeric constructs to identify a set of residues located in the structural proteins of the virus, namely a residue in prM and five residues in E, that account for the differences in growth kinetics, infectivity, and outcome of infection in animal models of the Cosmopolitan and Asian I types of DENV2. Insights into the mechanisms are only speculative and based on the construction of molecular homology models of the implicated proteins. Major concerns arise regarding the strength of data as the manuscript is missing figure legends describing the number of replicate experiments, data analysis, and the statistical tests applied.

**Response:** We sincerely apologize for the lack of figure legends. We have provided figure legends in the revised manuscript.

**Reviewer #2:**

**<Methods>**

Yes the study clearly articulates its hypothesis and is designed and executed to evaluate it.

**Response:** We appreciate this positive comment.

**<Results>**
Figures are good. I missed the figure legends.

**Response:** We sincerely apologize for the lack of figure legends. We provided figure legends in the revised manuscript.

< **Conclusions** >
Yes, the conclusions are for the most part supported by the data. See my specific points to see where they authors stray from that point.

**Response:** We appreciate this positive comment.

< **Summary and General Comments** >
In regions of southeast Asia, for example Thailand, there are circulating strains of all four dengue virus serotypes that expand and contract in the population. The authors of this manuscript examine the properties of two contemporary strains dengue 2 virus, the Asian-I genotype and the Cosmopolitan genotype, that circulate in Thailand. They demonstrate the superior growth in cell culture and in a mouse model of the Cosmopolitan strain. They go on to construct a series of chimeric Cosmopolitan – Asian viruses and map the regions and eventually some of the residues responsible for the enhanced replication phenotype. The authors conclude that prM-E need to be matched. They use the known structures to map the responsible residues and suggest that the Cosmopolitan strain may be less constrained in E-E interactions and this may result in enhanced replication. They also show that there are determinants in the nonstructural proteins NS1-NS2B from the Cosmopolitan strain also influence the enhanced replication. Collectively the manuscript is well written and the experiments are performed well.

**Response:** We appreciate this positive comment.

Specific comments:

I did not find figure legends.

**Response:** We sincerely apologize for the lack of figure legends. We have provided figure legends in the revised manuscript.

Lines 223-234: It would be best to directly compare the parental viruses with the cDNA-derived viruses to validate this statement.

**Response:** We are aware of the importance of this comment. Unfortunately, due to the regulations in Thailand and Japan, we had to perform experiments on the parental viruses in Thailand while the cDNA-derived viruses could only be handled in Japan. Therefore, we are unable to directly compare the parental viruses with the cDNA-derived viruses. We would greatly appreciate the understanding of the reviewer regarding this point.

Line 275: Define SRIP as not everyone will know this.

**Response:** We appreciate this comment. We defined SRIP at the first appearance of the term in the revised manuscript.

Line 358: A hydrogen bond would probably not contribute much to the stability of the E – E interactions.

**Response:** We appreciate this comment. We deleted the description on the hydrogen bond, and added more details on the predicted molecular models in the revised manuscript (lines 393-399).

Figure 5: In panel D the ACC virus has a larger focus size than CCC and yet CCC grows to higher titer (panel B). Can the authors address this?

**Response:** We appreciate this comment. In the revised manuscript, we described the results for the ACC virus more accurately (lines 286-288).

Line 370: This line is an overstatement to say that these 5 amino acids are responsible for replication of dengue virus. It needs to be restated to be more accurate.

**Response:** We apologize for this inaccurate conclusion. We reworded this sentence to be more accurate in the revised manuscript (lines 410-412). We also repeated the virus growth experiment in Figure 9, and showed the growth kinetics in revised Figure 9 to strengthen our conclusion on the effects of these five amino acids on virus growth.

Line 385: Given the results presented by the authors, why doesn’t the Cosmopolitan strain outcompete the Asian-I strain? They should discuss this.

**Response:** We appreciate this comment. There is a precedent in which a fast-growing lineage of dengue virus failed to replace a previous strain. We have cited this study (Pinheiro TM, *et al.,* 2018), and discussed the details in the revised manuscript (lines 434-440).

Line 441: Is there a clear connection between the strain of E and NS1 affecting replication? Perhaps this point should be discussed further if the authors can use their data to address this.

**Response:** We appreciate this comment. We cited a new reference that reported an interaction between E and NS1, and discussed the possible implications of their finding in the increased pathogenesis of the Cosmopolitan virus in the revised manuscript (lines 501-506).

**Reviewer #3:**

< **Summary and General Comments** >

The manuscript by Samune et al. investigates the differences in replication and pathogenesis of DENV2 strains isolated in Thailand in 2016-2017. They found that the NS1-NS2A-NS2B genes dictated pathogenesis in mice.

Major comments:
1. Were the rescued virus stocks sequenced? This is critical.

**Response:** We appreciate this comment. We determined the whole genome sequences of the generated virus stocks by next-generation sequencing. We found one unintended mutation in CCACC in Figure 4. Fortunately, exclusion of this CCACC from the analysis did not affect the conclusions of Figure 4. We described the sequencing results in the Results section of the revised manuscript (lines 274-276, 291-292, and 408-409).

2. Figure legends are missing. It was very hard to follow Fig. 4 without figure legends. What are the colors indicating? There are chimeras with A fragment of NS1-NS2A-NS2B with very high titers. Why would you want to highlight viruses with titers LOWER than A or HIGHER than C? This reflects viral incompatibilities. I would think you would want to highlight viruses with titers HIGHER than A or LOWER than C.

**Response:** We sincerely apologize for the lack of figure legends, which caused serious problems in interpreting our figures. We wanted to classify viruses into three categories: viruses showing similar titers to (or slightly higher than) the Cosmopolitan virus; viruses showing similar titers to (or slightly lower than) the Asian-I virus; and viruses showing titers between those of the two parental viruses. We would therefore like to keep the current color scheme in Figure 4. In the revised manuscript, we added a comment on two chimeras with Asian-I NS1, NS2A, and NS2B that showed high titers (lines 267-268). Since both of these chimeras carried Cosmopolitan structural proteins, these results also indicated that the genetic regions for structural proteins are important.

3. Which UTRs were used in the chimeras?

**Response:** We appreciate this comment. The 5’-UTR came from the 5’ proximal fragment F1, and the 3’-UTR came from the 3’ proximal fragment F5. To clarify this point, we revised panel A of Figure 2 to show that F1 and F5 contained the 3’- and 5’-UTRs, respectively, in the revised manuscript.

4. Focus forming assays on serum samples would be preferable to RNA levels.

**Response:** Unfortunately, there are no remaining serum samples of the viruses we analyzed in the present study. We would greatly appreciate it if the reviewers would allow us to publish our results without focus-forming assays of the serum samples.

5. In order to conclude that the 5 a.a. in the E protein interface are responsible for pathogenicity, mouse studies need to be performed. Alternatively, the language could be modified.

**Response:** We apologize for this inaccurate conclusion. We rewrote this sentence to be more accurate in the revised manuscript (lines 410-412).

We would greatly appreciate your reconsideration of our manuscript for publication.
