# Supplementary material for: Genetic regions affecting the replication and pathogenicity of dengue virus type 2
Source: PLoS Negl Trop Dis. 2024 Jan 8;18(1):e0011885. doi: 10.1371/journal.pntd.0011885 (PMC10798627; doi:10.1371/journal.pntd.0011885)
Supplement: Attachment — Submitted filename: ResponseToReviewers231130.docx [file pntd.0011885.s010.docx]

Our responses to the reviewers’ comments are as follows.

**Reviewer #1:**

**<Methods>**

(1) Figures 3 and 4. Comparison of virus growth of recombinant Asian-I and Cosmopolitan type recombinant dengue virus 2 in Figure 3B presents the means and standard deviation of technical replicates from a single experiment. Similarly, virus  titers in the supernatant of Vero cells infected with recombinant virus generated by CPER are presented as means and standard deviation of technical samples. Biological replicates are required to produce data with statistical significance. This is especially relevant as major hypotheses of the study, that is differences in the structural and NS1-2B coding sequences faster may account for the faster growth kinetics of the Cosmopolitan type virus, are based on the data presented in these two panels.

**Response:** Based on these suggestions, we repeated the experiments in Figs. 3 and 4 using independently-prepared virus stocks on parental and chimeric viruses for the structural and NS1, NS2A, and NS2B proteins. The results confirmed that these virus stocks possessed the same phenotypes as those shown in Figs. 3 and 4 and this information was added to the Results section (lines 277-281) and S2 Fig. of the revised manuscript.

(2) Figure 7. The experimental approach is problematic. Luciferase levels are not significantly different between constructs with the wild type or C709A mutant RdRp until 120 h, and even at this late time point the difference is still not statistically significant for one of the constructs (Gluc-CAAA vs. Gluc-CAAA (C709A), P = 0.1543). Statement made in lines 312-314 does not hold: "At first, luciferase activity is derived simply from the transfected CPER product, but it increases 48 h after transfection due to the Gluc-containing genome replication supported by non-structural proteins". Also, data in panel B are representative results of three independent experiments. As already mentioned, appropriate statistical analysis requires averaging of biological replicates. Finally, differences in luciferase levels between the recombinant constructs were also noted between RdRp mutants. Does the C709A mutant polymerase retain leaky activity?

**Response:** We appreciated these valuable comments. Based on these suggestions, we analyzed averages of the three independent experiments and found that all comparisons lacking statistical significance in the previous version of the manuscript showed statistically significant differences. We have added this information to the Results section of the revised manuscript (lines 331-353). We fully agree with the comment regarding the statement on lines 312-314 of the previous version of the manuscript and have modified this sentence in the revised manuscript (line 323). Regarding the C709A mutation, we have added one sentence on the possible leaky nature of this mutant in the revised manuscript (lines 357-359).

(3) Figure 10. As the figure presents the model of E-M-M-E heterotetramer, the term "envelope tetramer" used across the text should be avoided when referring to this structure. In line with previous comments, molecular modeling is not approapriate to compare binding between envelope proteins of the two types (lines 419-421 "Molecular modeling of the E protein tetramer suggested that the E protein dimer binds more loosely to neighboring E protein dimers in the Cosmopolitan virus than in the Asian-I virus").

**Response:** We apologize for our incorrect and misleading description of Figure 10. The structures shown in Figure 10 are two neighboring sets of E-M-M-E heterotetramer in a mature DENV particle and thus contain "envelope tetramer". To avoid any misunderstandings, we revised the Materials and Methods section (lines 198-200), the Results section (lines 411-423), the Discussion section (lines 452-455) and the title of Figure 10 to avoid using "envelope tetramer". Based on this comment, we also deleted our discussion on biding between two envelope dimers from the revised manuscript (line 455) and now merely describe the positions of the mutations in the structure.

(4) Figure 11. The authors mix up ionic and hydrogen bonds when referring to the proximity between hydrogen atom in the amino group of pr Lys 52 and oxygen in the carboxyl group pf E Glu 71. Lines 475-477: "Similar to the possible ionic bond between the E protein dimers of the Asian-I virus described above, these results indicated the possibility of the existence of an ionic bond between these residues in the Asian-I type (Fig. 11A, B)". The likely hydrogn bond formed between these two residues is not a ionic bond.

**Response:** Based on this comment, we deleted all of our description of ionic and hydrogen bonds (lines 514-511) from the revised manuscript.

**<Results>**
Although the results with the different approaches are aligned and point to the envelope protein as a determinant contributing to phenotypic differences between Cosmopolitan and Asian-I types, performing independent biological replicates where noted above can strengthen the analysis.

**Response:** Based on this comment, the results of independent biological replicates are now shown in S2 Fig. of the revised manuscript.

< **Conclusions** >
The authors have included a statement regarding the limitations of the CPER method referring to the rise of unintended mutations during PCR amplification. In the opinion of this reviewer, molecular models are over interpreted and conclusions and discussion referring to these models can be more concise.

**Response**: Based on this comment, we minimized the discussion of molecular modeling to be more concise.

< **Editorial and Data Presentation Modifications?**>

Figure legends were included in the revised version and references appropriately formatted.

**Response:** We appreciated this positive evaluation.

<**Summary and General Comments**>

In the revised version of the manuscript by Samune and colleagues the authors have responded to the criticisms of the reviewers point by point providing further experimentation and text edits including legends to figures. Overall, major comments were addressed.

**Response:** We appreciated this positive evaluation.

Specific comments regarding data analysis and limited value of the replicon assay are made above.

**Response:** As described above, we addressed these points in the revised manuscript.

**Reviewer #2:**

**<Methods>**

This is a revised manuscript. These questions were previously addressed.

**Response:** We appreciated this positive evaluation.

< **Summary and General Comments** >

This revised manuscript compares two current circulating strains of dengue 2 virus in Thailand and attempt to map the residues responsible for the differential replication and pathogenicity of these strains. The authors have done a reasonable job of responding to and revising their manuscript in response to reviewers’ comments and queries. The manuscript is clearer and the data is more robust in this revision.

**Response:** We appreciated this positive evaluation.

**Reviewer #3:**

< **Summary and General Comments** >

The authors have adequately addressed my concerns.

**Response:** We appreciated this positive evaluation.

We would greatly appreciate your reconsideration of our manuscript for publication.
