# Supplementary material for: Genetic regions affecting the replication and pathogenicity of dengue virus type 2
Source: PLoS Negl Trop Dis. 2024 Jan 8;18(1):e0011885. doi: 10.1371/journal.pntd.0011885 (PMC10798627; doi:10.1371/journal.pntd.0011885)
Supplement: Attachment — Submitted filename: ResponseToReviewers231220.docx [file pntd.0011885.s011.docx]

Our responses to the comments are as follows.

**Comments**

There are still issues with Figure 7. No new experiments are needed but modifications to the figure are required. While I understand the need to show "representative" experiments, it is clearer to show the combined averages of the 3 experiments in Figure 7. I believe you have this already in Supp Fig 3. Please make Figure 7B and C the combined averages of the 3 experiments with relevant statistics. Moreover, while you provided P values and statistics in the text, there is nothing in the figure legend I could find regarding which test you used. Please modify the figure legend to reflect the statistics as well.

**Response:** We appreciated these comments. Based on these comments, we showed the combined averages and standard deviations of the 3 independent experiments in Figure 7 and results of each of these 3 experiments are now in S3 Figure. We also revised the P values in the Results section (lines 324-331). We sincerely apologize for the lack of information on the statistical test used in Figure 7 and S3 Figure. We had used the Holm-Sidak method to correct multiple comparison, and this information was added the legends of the Figure 7 and S3 Figure of the revised manuscript.

We would greatly appreciate your reconsideration of our manuscript for publication.
